# Enhanced vasorin signaling mitigates adverse cardiovascular remodeling

**DOI:** 10.1002/agm2.12332

**Published:** 2024-06-19

**Authors:** Mingyi Wang, Kimberly Raginski McGraw, Robert E. Monticone, Roberta Giordo, Ali H. Eid, Gianfranco Pintus

**Affiliations:** ^1^ Laboratory of Cardiovascular Science, Intramural Research Program, National Institute on Aging, National Institutes of Health Biomedical Research Center (BRC) Baltimore Maryland USA; ^2^ Department of Biomedical Sciences University of Sassari Sassari Italy; ^3^ Department of Basic Medical Sciences, College of Medicine, QU Health Qatar University Doha Qatar

**Keywords:** adverse arterial remodeling, aging, calcification, fibrosis, inflammation, VASN

## Abstract

Arterial stiffening is a critical risk factor contributing to the exponential rise in age‐associated cardiovascular disease incidence. This process involves age‐induced arterial proinflammation, collagen deposition, and calcification, which collectively contribute to arterial stiffening. The primary driver of proinflammatory processes leading to collagen deposition in the arterial wall is the transforming growth factor‐beta1 (TGF‐β1) signaling. Activation of this signaling is pivotal in driving vascular extracellular remodeling, eventually leading to arterial fibrosis and calcification. Interestingly, the glycosylated protein vasorin (VASN) physically interacts with TGF‐β1, and functionally restraining its proinflammatory fibrotic signaling in arterial walls and vascular smooth muscle cells (VSMCs). Notably, as age advances, matrix metalloproteinase type II (MMP‐2) is activated, which effectively cleaves VASN protein in both arterial walls and VSMCs. This age‐associated/MMP‐2‐mediated decrease in VASN levels exacerbates TGF‐β1 activation, amplifying arterial fibrosis and calcification in the arterial wall. Importantly, TGF‐β1 is a downstream molecule of the angiotensin II (Ang II) signaling pathway in the arterial wall and VSMCs, which is modulated by VASN. Indeed, chronic administration of Ang II to young rats significantly activates MMP‐2 and diminishes the VASN expression to levels comparable to untreated older control rats. This review highlights and discusses the role played by VASN in mitigating fibrosis and calcification by alleviating TGF‐β1 activation and signaling in arterial walls and VSMCs. Understanding these molecular physical and functional interactions may pave the way for establishing VASN‐based therapeutic strategies to counteract adverse age‐associated cardiovascular remodeling, eventually reducing the risk of cardiovascular diseases.

## Graphical abstract

Vasorin (VASN)‐mediated arterial proinflammatory signaling pathway: (1) inhibits transforming growth factor‐beta1 (TGF‐β1) fibrotic signaling pathway; (2) negatively affects angiotensin II (Ang II)‐TGF‐β1 fibrotic and inflammatory activated MMP pathway; (3) exerts antiaging effects via the blockade of Ang II and matrix metalloproteinase type II activation pathway; and (4) miR146a modulates VASN expression and effects. VANS signaling potentially mitigate adverse cardiovascular remodeling and retards cardiovascular disease.

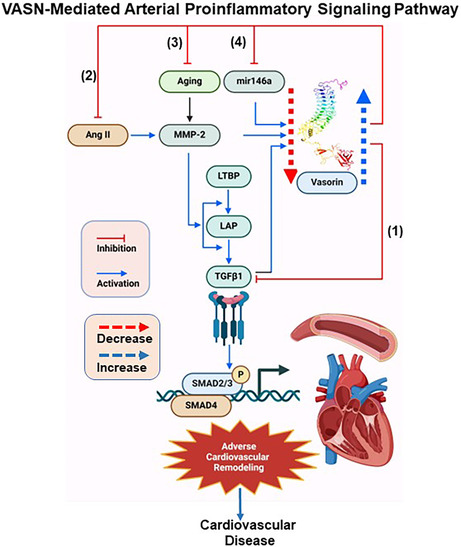

## 1 INTRODUCTION

The global increase in the aging population is causing a surge in the incidence of cardiovascular disorders such as hypertension, atherosclerosis, heart failure, and vascular associated cognitive decline and dementia.^1^ A growing body of evidence indicates cardiovascular stiffening as one of the major risk factors for the onset and progression of these age‐associated cardiovascular disorders.^2^, ^3^, ^4^ Indeed, the wall thickening, fibrosis, and calcification observed in the aging cardiovascular system lay the foundation for the development of cardiovascular stiffening and associated diseases.^2^, ^3^, ^4^, ^5^, ^6^

In the aging cardiovascular system, a loss of balance between the pro‐fibrotic signaling molecule, transforming growth factor‐beta1 (TGF‐β1), and the anti‐fibrotic signaling molecule Vasorin (VASN), may play a determinant role in the initiation and progression of cardiovascular thickening, collagen deposition, and calcification, prompting age‐associated adverse cardiovascular remodeling and triggering age‐associated diseases.^4^, ^7^, ^8^, ^9^, ^10^, ^11^ In fact, this imbalance has been shown to significantly contribute to cardiovascular fibrosis and stiffening with advancing age, development, or mechanical and metabolic insults.^4^, ^7^, ^9^, ^10^, ^12^

During aging, both TGF‐β1 and angiotensin II (Ang II) signaling increase in the cardiovascular system.^13^, ^14^, ^15^, ^16^, ^17^ In this context, TGF‐β1, an Ang II downstream signaling molecule and a cleaved product of the matrix metalloproteinase type II (MMP‐2), plays a well‐established role in mediating cellular phenotypic shifts and matrix deposition, ultimately affecting cardiovascular remodeling.^13^, ^14^, ^15^, ^16^, ^17^ Of note, such a signaling network within the cardiovascular system is tightly modulated by VASN.^10^, ^16^, ^18^, ^19^

In this minireview, we delve into the role of VASN in modulating Ang II and TGF‐β1 molecualr siganling; and in the structural and functional remodeling of the cardiovascular system during aging and age‐associated diseases. Understanding how VASN operates in these processes may open a new window for therapeutic interventions to prevent and treat cardiovascular aging and associated diseases.

## 2 VASORIN PROTEIN EXPRESSION IN ARTERIAL WALLS AND VSMCs

**FIGURE 1:**
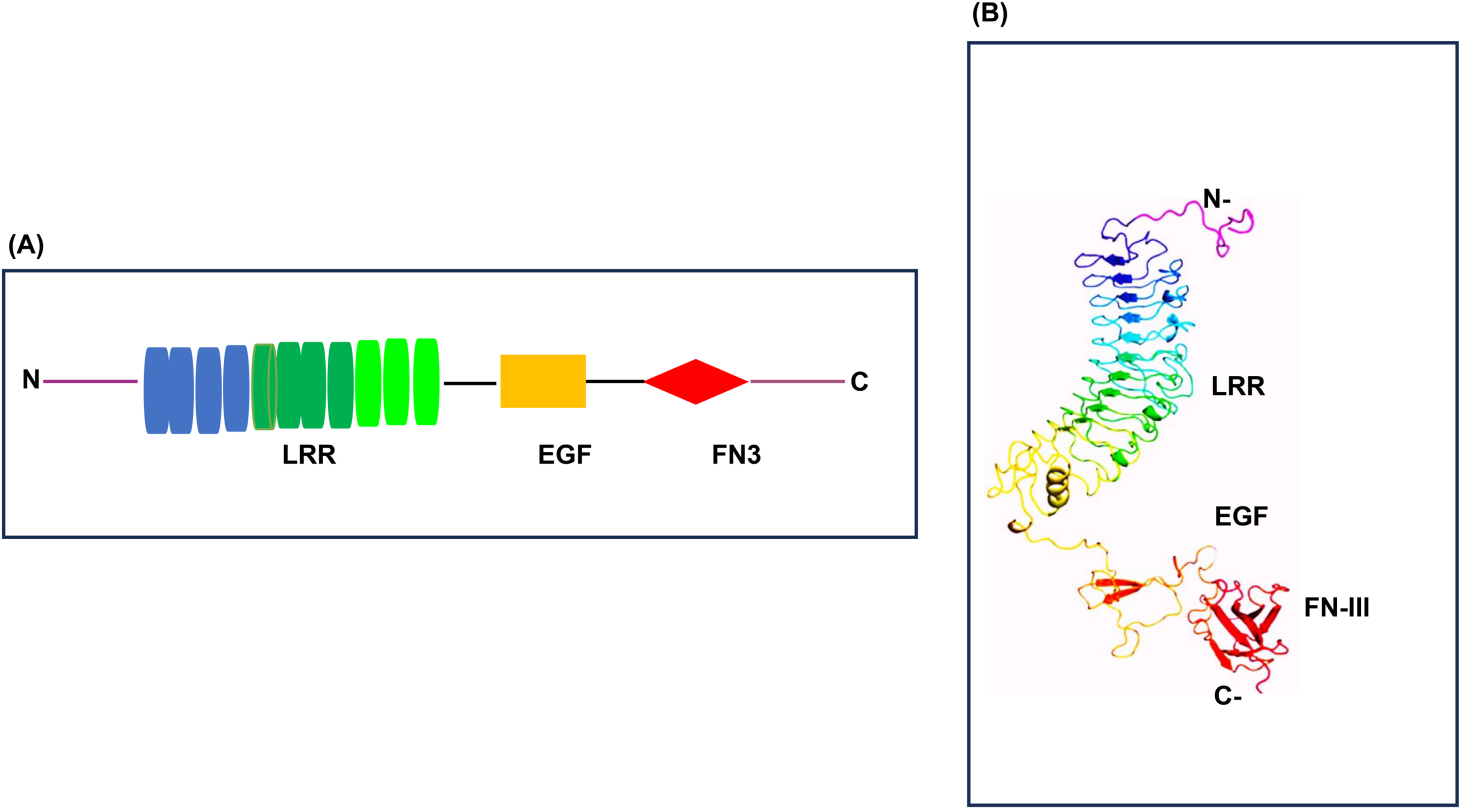
VASN structure. (A) VASN structural model modified from Ikeda et al.^7^ (B) VASN 3D structure modified from Bonnet et al.^20^ LRR, leucine‐rich repeat motif; EGF, epidermal growth factor‐like domain; FN3, fibronectin‐3 like motif; N, N‐terminus; C, C‐terminus.

**FIGURE 2:**
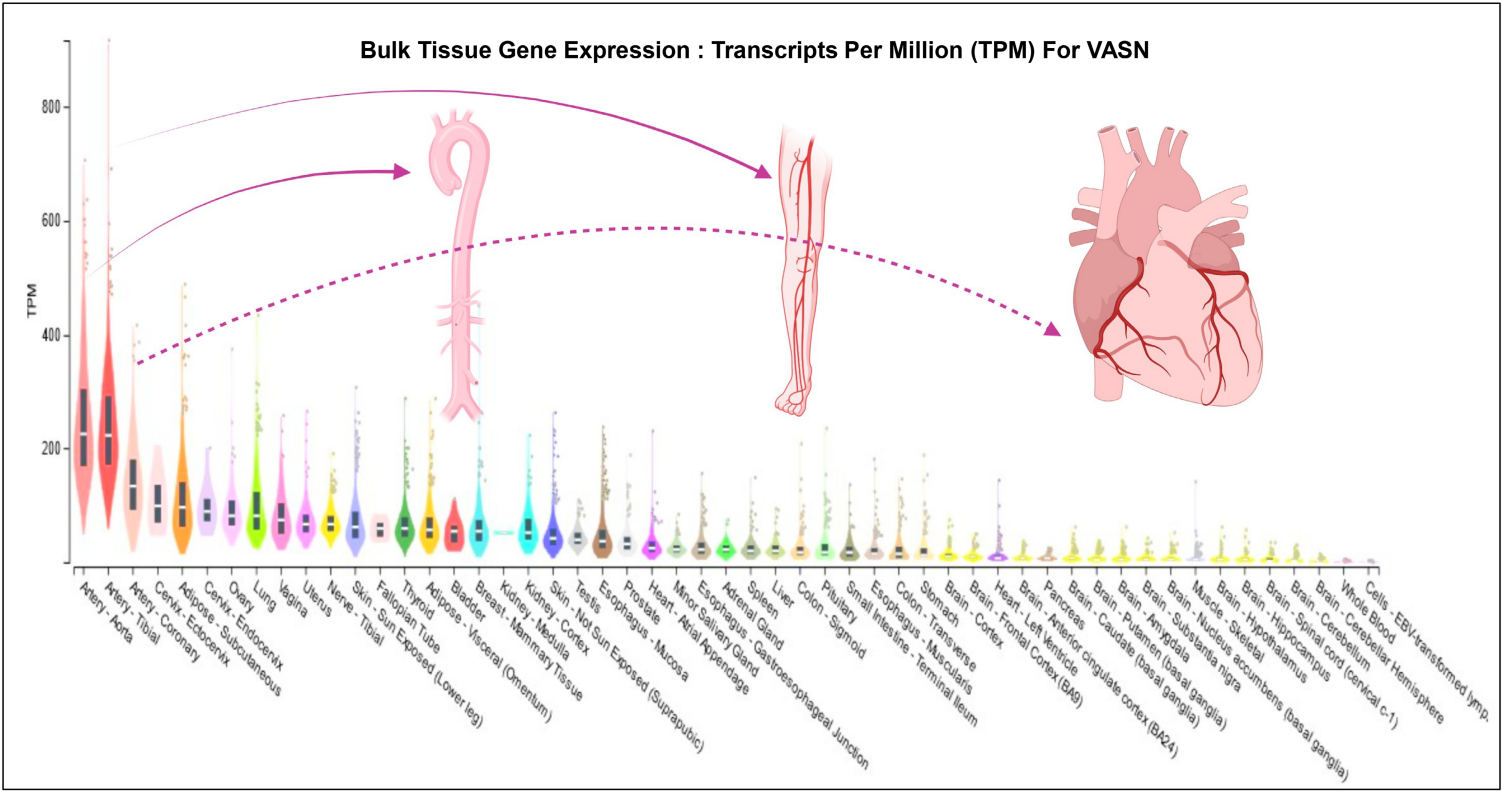
VASN expressed in human tissue: Bulk tissue gene expression for VASN (ENSG00000168140.4) modified from the data source: GTEx analysis release V8 (dbGaP Accession phs000424.v8.p2), located at https://gtexportal.org/home/gene/VASN; and generated by BioRender (http: www.biorender.com).

**FIGURE 3:**
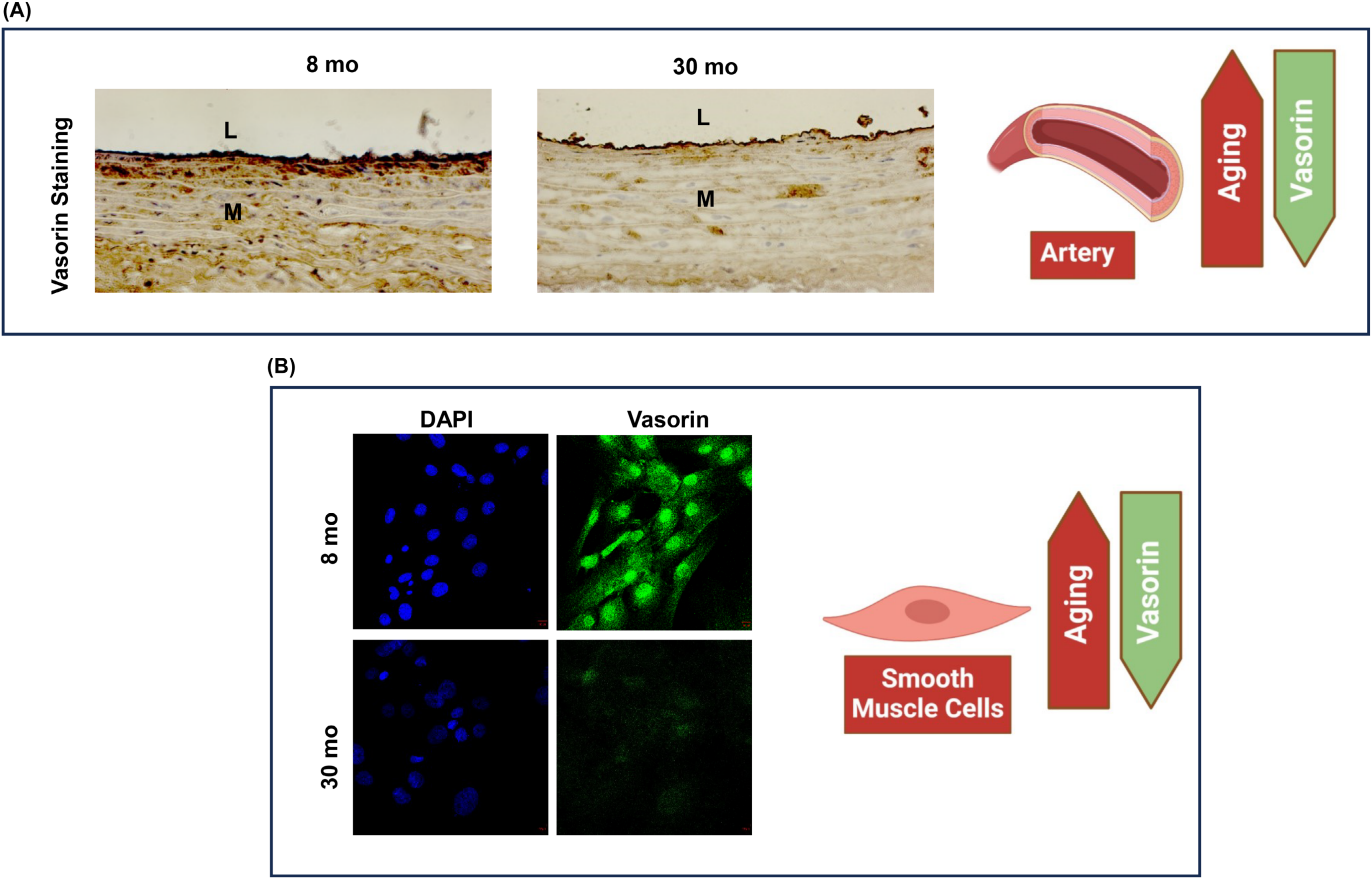
VASN staining in aging arterial walls and VSMCs. VASN protein in arterial walls (A. brown color, upper panels) and VSMCs (B. green color, lower panels) modified from Pintus et al.^10^ L, lumen; M, media.

VASN, known as an anti‐tissue necrosis factor alpha‐induced apoptosis or slit‐like‐2 protein, belongs to the classic type I cellular membrane protein category.^7^, ^10^ It features 10 tandem arrays of leucine‐rich repeat motifs (LRR), an epidermal growth factor‐like motif (EGF), and a fibronectin type III‐like motif (FN) at its extracellular domain (Figure 1A). Of note, VASN physically traps TGF‐β1 through its extracellular domains, specifically the LRR, EGF‐like, and FN forming a pocket, but the precise amino acid sequences involved in this interaction remain elusive (Figure 1B).^7^, ^20^ This protein is highly glycosylated and is predominantly expressed in arterial walls, including the aorta, tibial, and coronary walls (Figure 2: https://gtexportal.org/home/gene/VASN).^7^, ^8^, ^9^, ^10^, ^11^, ^12^, ^20^, ^21^, ^22^ VASN mRNA and protein exhibit high levels of expression in vascular smooth muscle cells (VSMCs) within aortic walls during mice embryonic development.^22^ On the contrary, the VASN expression is significantly lower in premature klotho‐hypomorphic mice with a short lifespan of 8–9 weeks.^9^ Furthermore, transcription and translational levels of VASN are reduced in adult mice with injured arteries, coinciding with increased neointima formation.^7^ Notably, VASN mRNA levels are markedly downregulated in old compared to young rat aortae, and VASN protein levels are also significantly decreased in old versus young rat aortae (Figure 3, upper panels).^10^ In parallel, VASN mRNA and protein in primary cultured old VSMCs are markedly down egulated compared to young cells (Figure 3, lower panels).^10^ Importantly, abundant VASN expression has been detected in young, healthy nonhuman primates and human aortic walls.^10^ The above findings suggest that the VASN expression is closely associated with cardiovascular development, injury, and aging.

### 2.1 Vasorin physical and functional interaction with TGF‐β1 and MMP‐2 in arterial walls and VSMCs

#### 2.1.1 VASN and MMP‐2

**FIGURE 4:**
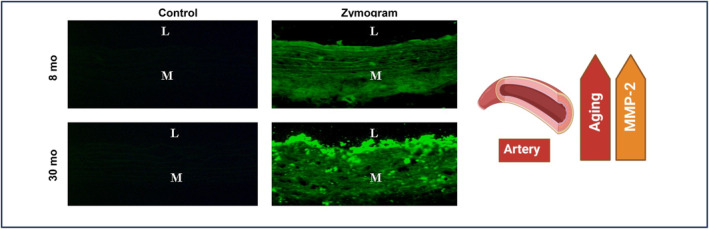
Activated MMP‐2 increases in aging arterial walls. Aging increases activated MMP‐2 determined by in situ zymography (Green color) modified from Wang et al.^17^ L, lumen; M, media.

Age markedly increases arterial MMP‐2 activation (Figure 4). It has been reported that VASN is a substrate for the activated zinc‐ and calcium‐dependent gelatinase MMP‐2.^10^, ^18^, ^19^ MMP‐2 activation is driven by the membrane type‐matrix metalloproteinase 1 (MT‐MMP1), a potent MMP‐2 activator, and counteracted by the tissue inhibitor of metalloproteinases 2 (TIMP2), an effective MMP‐2 inhibitor.^23^ Notably, a coordinated modulation of MT‐MMP1, MMP‐2, and TIMP2 occurs in the arterial wall in response to aging or injury.^14^, ^24^, ^25^

In this regard, a growing body of evidence indicates a significant increase in MMP‐2 activation in aging VSMCs and arterial walls across various species, including mice, rats, nonhuman primates, and human aortic samples.^4^, ^14^, ^25^, ^26^ Moreover, arterial MT‐MMP1 levels rise while TIMP2 levels decline with aging in these species.^4^, ^14^, ^25^, ^26^ Similarly, protein and activity of both MT‐MMP1 and MMP‐2 markedly increased in injured arterial walls, while TIMP2 significantly decreased.^24^

Interestingly, data collected in previous studies indicated that both aging‐ and injury‐associated reduction of VASN protein is likely attributed to post‐translational modifications exerted, at least in part, by an MMP‐2‐mediated enzymatic cleavage, since VASN is a degradative substrate of activated MMP‐2.^7^, ^10^, ^18^, ^19^ Moreover, aging has been shown to be linked to increased MMP‐2 activation and decreased VASN in the arterial wall and VSMCs, further supporting this novel conception.^10^

Indeed, activated MMP‐2 dose‐dependently cleaves the VASN protein in vitro and ex vivo in monkey and human aortic tissues.^10^ Noteworthy, preventing VASN cleavage with the MMP inhibitor PD166793 has been reported to block the adverse age‐associated TGF‐β1 fibrotic signals in rat aortic walls and cells.^10^

### 2.2 VASN and TGF‐β1

**FIGURE 5:**
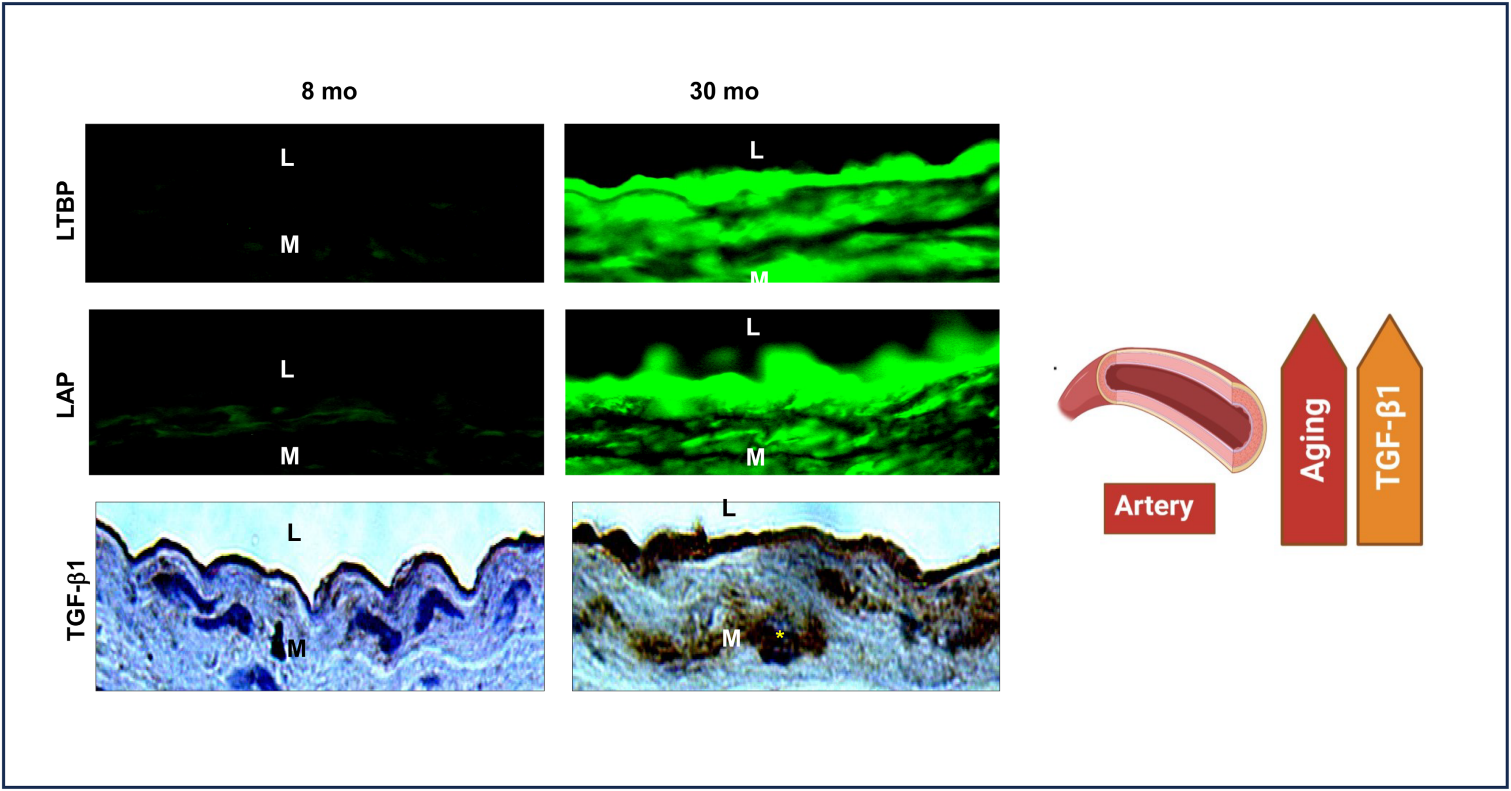
TGF‐β1 increases in aging arterial walls. Aging increases expression of the precursor proteins: LTBP1 and LAP (green color), and activated TGF‐β1 protein (brown color), modified from Wang et al.^17^ L, lumen; M, media. LTBP, latent TGF binding protein; LAP, latent associated protein; TGF‐β1, transforming growth beta‐1.

Our group reported that aging decreases VASN while increasing TGF‐β1 precursors such as the latent TGF binding protein‐1 (LTBP‐1), latent associated protein (LAP), and active TGF‐β1 in arterial walls (Figure 5).^10^, ^17^ Moreover, our own along with other studies have documented that VASN forms a physical interaction with TGF‐β1 and functionally hinders TGF‐β1 downstream signaling, including Suppressor of Mothers against Decapentaplegic‐2/3 (SMAD‐2/3) phosphorylation, collagen production, and calcification.^7^, ^9^, ^10^ This inhibition occurs through the prevention of TGF‐β1 from accessing its receptors, TGF β receptor type I & II, situated on the VSMC surface and within mouse and rat vascular walls.^7^, ^9^, ^10^ Moreover, exposing human VSMCs to TGF‐β1, per se, eventually results in a significant reduction of VASN and a synergistic increase in TGF‐β receptor type 1, known as actin receptor‐like kinase 5 (ALK5), and its downstream molecule p‐SMAD2.^9^

## 3 VASORIN ALLEVIATES ANG II SIGNALING IN ARTERIAL WALLS AND VSMCs

The aging process has been reported to exacerbate Ang II‐mediated signaling, decrease VASN protein, and eventually modify TGF‐β1‐mediated signals within the arterial wall.^14^, ^15^, ^16^, ^27^, ^28^, ^29^ During aging, the octapeptide Ang II and its AT 1 receptor expression are upregulated, while its AT 2 receptor is downregulated in arterial walls.^13^, ^14^, ^16^, ^28^, ^29^ This age‐induced imbalance is closely associated with heightened sympathetic autonomic nerve activity and chronic cyclic mechanical strain.^14^, ^30^ Indeed, the sympathetic neurotransmitter norepinephrine and its α‐receptor expression are upregulated during aging, leading to arterial wall inflammation, fibrosis, and calcification.^31^, ^32^ A response to this aging‐triggered inflammation and matrix events is the increase in Ang II protein abundance, the AT1 receptor upregulation, and the AT2 receptor downregulation.^13^, ^29^ In addition, the age‐associated rise in systolic blood and pulse pressure elevates the chronic arterial cyclic mechanical strain force, potentially upregulating the expression of both the Ang II peptide and the AT1 receptor.^13^, ^14^, ^15^, ^16^, ^28^, ^29^, ^33^

This age‐associated increment of Ang II‐mediated signals is intricately linked to the decline in VASN expression in both arterial walls and VSMCs.^10^ Interestingly, similar to the aging process, Ang II, per se, administration is able to reduce VASN protein expression in both arterial walls and VSMCs in an AT1 receptor‐mediated fashion.^10^ Conversely, blocking the AT1 receptor signaling increases the expression of VASN in VSMCs.^10^ Notably, Ang II reduces VASN protein levels primarily by increasing MMP‐2 cleavage activity in VSMCs and the arterial wall.^10^ Moreover, similar to aging, infusion of Ang II in young rats significantly activates MMP‐2 in the arterial wall,^13^, ^16^ and exposure of young VSMCs to Ang II also markedly activates MMP‐2.^10^, ^14^ Since Ang II has been reported to activate TGF‐β1,^13^, ^16^ it is reasonable to conclude that Ang II induces VSMCs or arterial wall fibrosis likely resulting mainly from a disruption of the VASN‐TGF‐β balance in an MMP‐2‐mediated manner.^10^

## 4 VASORIN MODIFIES THE VSMC PHENOTYPE

Ang II, TGF‐β1, MMP‐2, and VASN are pivotal signaling molecules which not only modulate extracellular matrix (ECM) remodeling but also influence VSMCs phenotype.

### 4.1 VSMC secretion

VSMCs, the predominant cell type in arterial walls, become activated and inflamed, transitioning from a quiescent to a synthetic phenotype with aging and age‐associated diseases.^4^, ^23^ These synthetic VSMCs secrete large amounts of inflammatory molecules such as MMP‐2 and ECM, particularly collagen.^4^, ^23^ Compelling data indicated that this age‐associated collagen accumulation is due mainly to the augmented Ang II signaling, coupled with the activation of MMP‐2 and TGF‐β1 signaling.^14^, ^15^, ^16^, ^25^, ^34^ It is well‐known that collagen deposition in the thickened aortic wall is a characteristic histologic feature of arterial aging and mechanical injury.^14^, ^15^, ^16^, ^25^, ^34^ Importantly, age‐associated secretion of VSMCs facilitates cellular phenotypic shifts and extracellular matrix modifications such as fibrosis and calcification.^4^, ^23^

### 4.2 VSMC proliferation

Increased proliferation of VSMCs is associated with various vascular remodeling processes and diseases, including age‐associated intimal thickening, atherosclerosis, and in‐stent restenosis.^5^, ^27^, ^35^, ^36^, ^37^, ^38^ One main factor contributing to phenotype switch of VSMCs from quiescent to proliferative is the activation of inflammatory signaling cascades, which lead to the downregulation of crucial contractile structural and cytoskeletal proteins.^4^, ^5^, ^23^, ^27^ In this context, the smooth muscle cell‐specific protein VASN is emerging as a novel regulator of VSMC proliferation and differentiation both in vitro and in vivo.^7^, ^8^, ^9^, ^10^, ^21^, ^22^

Indeed, VSMCs lacking VASN exhibit reduced expression of VSMCs differentiation‐associated marker molecules, such as smoothelin and calponin, facilitating proliferation.^21^ In contrast, overexpression of VASN in VSMCs in vitro significantly reduces serum‐induced proliferation.^21^ Mechanistically, VASN directly binds to the epidermal growth factor receptor (EGFR), inhibiting EGFR phosphorylation and a subsequent proliferative event.^21^

Additionally, VASN has been identified as a direct target of miRNA‐146a, whose expression is upregulated following wire‐induced injury in vivo.^21^ In this context, inhibiting miRNA‐146a using specific antisense nucleotides (LNAs) enhances the VASN expression in VSMCs in vivo.^21^ Moreover, restoring the VASN expression through miR‐146a inhibition prevents VSMC de‐differentiation and proliferation following vascular injury, thereby preventing neointima formation.^21^ These findings suggest that miR‐146a tightly regulates the VASN expression, making it a cell‐specific regulator of VSMC differentiation and proliferation. In this light, targeting miR‐146a‐VASN may represent a novel, effective, and cell‐specific approach to prevent neointima hyperplasia.

### 4.3 VSMC migration/invasion

VSMC migration and invasion are key cellular events in age‐associated diffuse intimal thickening or injury‐induced neointimal formation triggered by proinflammatory molecules such as MMP‐2.^23^, ^24^, ^27^, ^35^ Invasion and migration of VSMCs are influenced by MMP‐2 activation, which is progressively activated with advancing age and is an essential modulator of these cellular events.^23^ Notably, the ability of invasion and migration of VSMCs isolated from old rats are significantly higher than those of cells isolated from young animals; and these age‐related effects are substantially reduced by the MMP‐2 inhibitor GM6001.^23^

Intriguingly, exposing young VSMCs to Ang II enhances their invasive capacity to levels observed in untreated older cells, and this effect was significantly diminished by an Ang II blocker.^16^, ^23^ In this context, increased infiltration of intimal VSMCs is pivotal in age‐associated arterial remodeling and is closely linked to the activation of the Ang II/MMP‐2 signaling pathway.^16^, ^23^ In fact, Ang II increases MMP‐2 activation and facilitates the rapid transition of VSMCs from a contractile, differentiated phenotype to a synthetic, dedifferentiated, invasive phenotype.^10^, ^13^, ^16^, ^28^ Of note, VASN overexpression or VASN protein administration inhibits Ang II‐associated MMP‐2 activation and VSMCs invasion during aging.^10^ These findings underscore the role of VASN in modulating the aging/Ang II‐associated MMP‐2 activation and VSMCs invasive behavior.

### 4.4 VSMC collagen deposition

By interacting with TGF‐β1, VASN can mimic the action of Ang II AT1 receptor antagonist, alleviating age‐associated pro‐fibrogenic collagen production in VSMCs.^10^ Indeed, VASN avidly binds to TGF‐β1, subsequently blocking the activation of TGF‐β receptors type I and II, along with their downstream signals SMAD‐2/3 and collagen secretion.^7^, ^9^, ^10^ Noteworthy, VASN overexpression in aged VSMCs results in decreased TGF‐β1 downstream signaling, including SMAD‐2/3 phosphorylation and collagen I production, resembling the effects exerted by the AT1 receptor antagonist Losartan.^10^ Moreover, VASN overexpression in young VSMCs effectively blocks Ang II‐induced increases in TGF‐β1 signaling, including SMAD‐2/3 phosphorylation and collagen I deposition.^10^ Importantly, VASN‐treated aged VSMCs showed substantially lower levels of TGF‐β1 downstream molecules such as p‐SMAD‐2/3, even though the levels of activated TGF‐β1 remained constant.^10^ These findings support the hypothesis that VASN alleviates TGF‐β1 signaling by obstructing its access to the TGF‐β receptors. They also suggest that VASN physically traps TGF‐β1, mimicking the action of the AT1 antagonist Losartan, and mitigating the Ang II‐associated fibrotic effects in aged arterial walls and VSMCs.

### 4.5 VSMC calcification

By binding TGF‐β1 and inhibiting the TGF‐β receptor signaling, VASN also plays a crucial role in regulating osteo−/chondrogenic trans‐differentiation and VSMCs calcification.^9^ Indeed, in vivo, aortic VASN expression was reduced in the hyperphosphatemia klotho‐hypomorphic mouse model, a premature age‐ or chronic kidney disease (CKD)‐related vascular calcification model.^9^ In vitro, VASN treatment suppressed TGF‐β1 signaling and blocked osteo−/chondrogenic trans‐differentiation and VSMCs calcification in pro‐calcifying conditions such as in a medium with a high concentration of phosphate.^9^

In a recent study, Luong et al. treated primary human VSMCs with recombinant human TGF‐β1 in the presence or absence of recombinant human VASN or VASN gene silencing.^9^ They found that TGF‐β1 treatment downregulated VASN mRNA expression in human VSMCs while VASN treatment inhibited TGF‐β1downstream molecule SMAD2 phosphorylation and TGF‐β1‐downstream target genes SRY‐box transcription factor 2 (SOX2), Runt‐related transcription factor 2 (Runx2), matrix metalloproteinase 13 (MMP13), collagen, and alkaline phosphatase (ALP) expression.^9^ Importantly, VASN treatment was able to completely abolish the TGF‐β1‐induced increase of ALP activity, a key enzyme for the development of ectopic calcification in the arterial wall.^9^ Notably, VASN treamentr also mitigated TGF‐β1‐induced osteo−/chondrogenic trans‐differentiation of VSMCs while VASN silenceing augmented these effects.^9^ Additionally, phosphate treatment reduced VASN mRNA expression in human VSMCs, further increasing its pro‐calcificating capability.^9^ VASN treatment did not affect phosphate‐induced TGF‐β1 expression, but it blunted phosphate‐induced TGF‐β1 signaling, osteo−/chondrogenic trans‐differentiation, and calcification of human VSMCs; and VASN silencing aggravated osteoinduction in human VSMCs cultured in high phosphate conditions.^9^ These findings suggest that VASN treatment inhibits calcification of VSMCs induced by phosphate mainly via a blockade of TGF‐β1 downstream calcifying signaling rather than its expression levels.

In addition, urea treatment markedly increases the MMP‐2 activity while decreases the VASN expression in human microvascular endothelial cells, promoting endothelial‐to‐mesenchymal transition, which is a potential mechanism underlying cardiovascular calcification in CKD with urea accumulation.^12^

Taken together, these findings position VASN as a novel key regulator of VSMC calcification and a potential therapeutic target for vascular calcification during aging or CKD.

### 4.6 Vasorin in cardiovascular remodeling and disease

**FIGURE 6:**
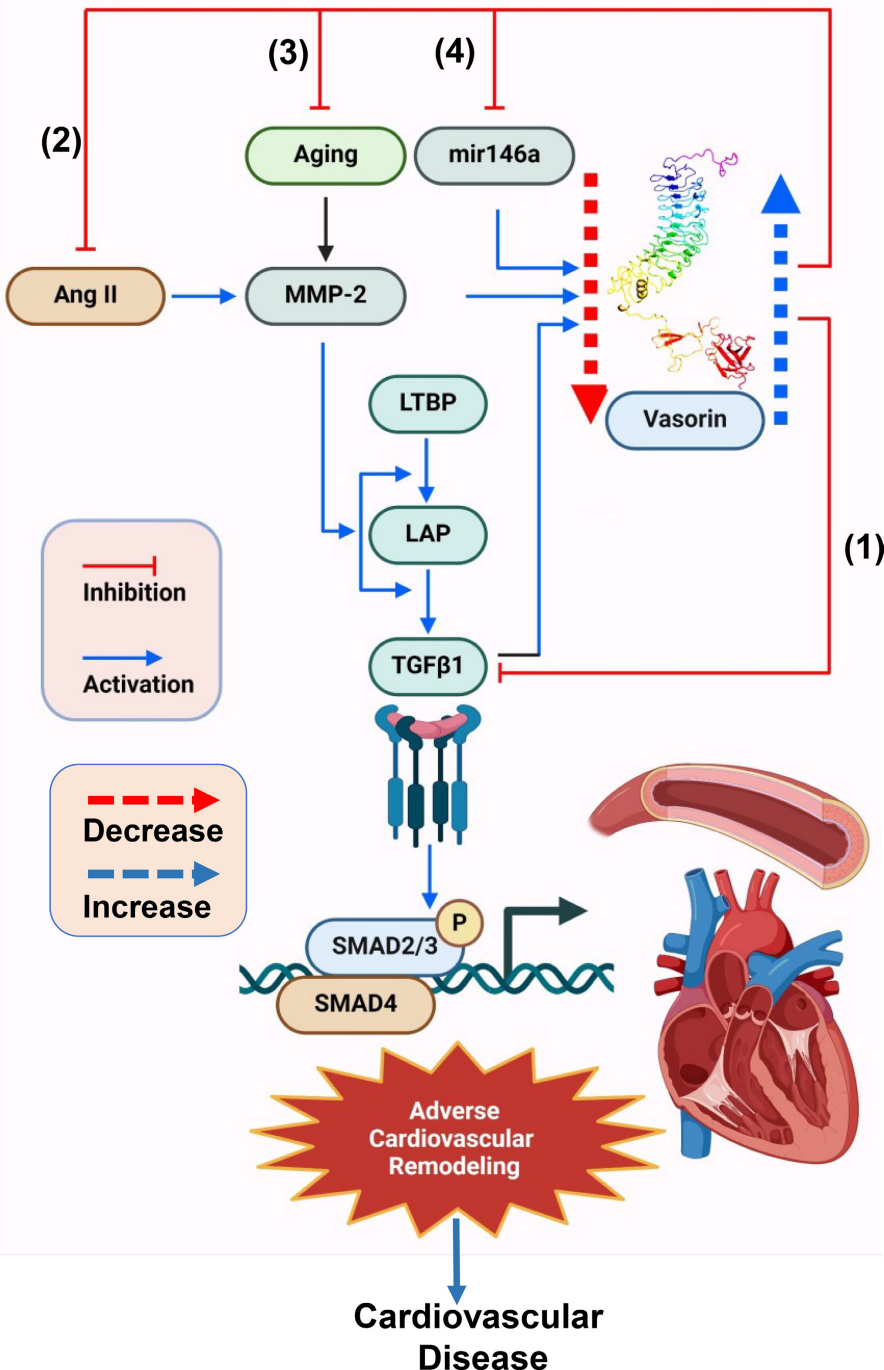
VASN‐Mediated arterial proinflammatory signaling pathway. (1) VASN directly inhibits TGF‐β1 fibrotic signaling pathway; (2) VASN negatively affects Ang II‐TGF‐β1 fibrotic and inflammatory activated MMP pathway: (3) VASN exerts anti‐aging effects via the blockade of Ang II and MMP‐2 activation pathway; and (4) miR146a modulate the VASN expression and proliferation of VSMCs. These VASN mediated signaling pathways collectively mitigate arterial VSMC migration, proliferation, fibrosis, and calcification, a cellular matrix foundation of adverse cardiovascular remodeling: arterial thickening/stiffening and cardiac hypertrophy/stiffening; and eventually alleviating cardiovascular disease such as hypertension, atherosclerosis, and heart failure. Ang II, angiotensin II; LTBP, latent TGF binding protein; LAP, latent associated protein; SMAD2/3/4, suppressor of mothers against decapentaplegic‐2/3/4; TGF‐β1, transforming growth factor beta‐1.

The currents data suggest that VASN can counteract arterial proinflammatory signaling pathways: VASN directly interferes with the TGF‐β1 signaling pathway, attenuating its profibrotic signals within the arterial wall; VASN alleviates Ang II/TGF‐β1/MMP‐2‐mediated arterial inflammation and fibrosis with advancing age; and miR146a regulates the VASN expression and its downstream effects, inhibiting the proliferation of VSMCs. Collectively, the VASN‐modulated signaling pathways appear to play a crucial role in mitigating adverse cardiovascular remodeling, including arterial thickening/stiffening and cardiac hypertrophy/stiffening, potentially preventing the development of cardiovascular diseases such as hypertension, atherosclerosis, and heart failure (Figure 6).

## 5 VASORIN IN ADVERSE ARTERIAL WALL REMODELING AND ARTERIAL DISEASE

Mounting evidence demonstrates that VASN effectively counteracts adverse arterial remodeling such as arterial restenosis and calcification.^7^, ^9^ Notably, tissue VASN protein levels exhibit a negative correlation with the degree of post‐injury arterial restenosis in an experimental animal model,^7^ and low circulating VASN levels are closely associated with the prevalence and severity of aortic valve calcification in humans.^39^ In line with these findings, VASN overexpression significantly alleviates neointima thickening and calcification,^7^, ^9^, ^21^ and interventions aimed at modulating the VASN expression, such as administration of the immunosuppressant FK778 or miR‐146a inhibition, show promise in reducing coronary restenosis and arterial neointima formation.^21^, ^37^ Importantly, VASN potentially modulates arterial development and functions such as blood pressure, blood filling, and blood flow velocity.^8^, ^10^, ^22^, ^40^ In addition, previous findings suggest that VASN levels in the blood or vascular tissue are significantly associated with the onset and development of hypertension and atherosclerosis.^41^, ^42^, ^43^ Notably, VASN knockout mice display significant arterial dysfunction, characterized by decreased systolic blood pressure, impaired contractile response to Ang II or phenylephrine, and endothelium‐dependent relaxation damage, ultimately resulting in the demise of all mice within 3 weeks after birth.^8^, ^11^ These findings highlight the crucial role of VASN in maintaining vascular homeostasis, and its potential role in the development of hypertension, arterial restenosis, and atherosclerosis.

## 6 VASORIN IN CARDIAC REMODELING AND HEART FAILURE

Recent evidence expands the role of VASN beyond vascular remodeling, implicating it in adverse cardiac remodeling.^11^ Indeed, Sun et al reported that VASN knockout mice exhibit markers of myocardial metabolic abnormalities and myocardial injury such as elevated levels of aspartate aminotransferase, homocysteine, and lactate dehydrogenase.^11^ The absence of VASN also contributed to increased heart weight and cardiomyocyte size, indicating cardiac hypertrophy.^11^ Furthermore, the lack of VASN led to a significant increase in mitochondrial breakdown and death of cardiomyocytes, resulting in heart dysfunction, characterized by elevated levels of B‐type natriuretic peptide and myosin heavy chain.^8^, ^11^ It is well known that the death and hypertrophy of cardiomyocytes are the fundamental cellular mechanism underlying cardiac failure.

## 7 CONCLUSION AND FUTURE OUTLOOK

Large amount of ever‐growing evidence indicates that VASN is a key signaling molecule in the development of adverse cardiovascular remodeling and associated diseases (Figure 6). VASN is not only linked to acute arterial injury but also plays a crucial role in the process of age‐associated chronic adverse cardiovascular remodeling by modulating signaling cascades triggered by Ang II, MMP‐2, and TGF‐β1. In response to Ang II, the levels of VASN decrease due mainly to an MMP‐2 cleavage. Consequently, this VASN decrease amplifies the increase of TGF‐β1 signaling induced by Ang II, potentially contributing to cardiovascular inflammation, fibrosis, and calcification. Thus, maintaining appropriate levels of arterial and cardiac VASN could be an innovative therapeutic strategy to prevent adverse cardiovascular remodeling elicited by inflammation, fibrosis, and calcification, as well as associated cardiovascular diseases such as hypertension, atherosclerosis, and heart failure.

## AUTHOR CONTRIBUTIONS

M. W.: concepting, supervising, writing original draft, revising, and editing. K. R. M.: revising and editing. R. M.: revising and editing. R. G.: revising and editing. A. H. E.: revising and editing. G. P.: concepting, writing, revising, and editing. All authors provided final approval for publication.

## FUNDING INFORMATION

This research was supported by the Intramural Research Program of the National Institute on Aging, National Institutes of Health.

## CONFLICT OF INTEREST STATEMENT

None.

## ETHICS STATEMENT

Not applicable.

## References

[1] Martin SS , Aday AW , Almarzooq ZI , et al. 2024 heart disease and stroke statistics: a report of US and global data from the American Heart Association. Circulation. 2024;149(8):e347‐e913. doi:10.1161/cir.0000000000001209 38264914 PMC12146881

[2] AlGhatrif M , Lakatta EG , Morrell CH , et al. Dilated hypertrophic phenotype of the carotid artery is associated with accelerated age‐associated central arterial stiffening. Geroscience. 2023;45(2):1001‐1013. doi:10.1007/s11357-022-00699-w 36520341 PMC9886763

[3] Gavish B , Bursztyn M , Thijs L , et al. Predictive power of 24‐h ambulatory pulse pressure and its components for mortality and cardiovascular outcomes in 11848 participants recruited from 13 populations. J Hypertens. 2022;40(11):2245‐2255. doi:10.1097/hjh.0000000000003258 35950994 PMC10366954

[4] Wang M , Monticone RE , McGraw KR . Proinflammation, profibrosis, and arterial aging. Aging Med (Milton). 2020;3(3):159‐168. doi:10.1002/agm2.12099 33103036 PMC7574637

[5] Aherrahrou R , Baig F , Theofilatos K , et al. Secreted protein profiling of human aortic smooth muscle cells identifies vascular disease associations. Arterioscler Thromb Vasc Biol. 2024;44(4):898‐914. doi:10.1161/ATVBAHA.123.320274 38328934 PMC10978267

[6] Roth L , Dogan S , Tuna BG , et al. Pharmacological modulation of vascular ageing: a review from VascAgeNet. Ageing Res Rev. 2023;92:102122. doi:10.1016/j.arr.2023.102122 37956927

[7] Ikeda Y , Imai Y , Kumagai H , et al. Vasorin, a transforming growth factor beta‐binding protein expressed in vascular smooth muscle cells, modulates the arterial response to injury in vivo. Proc Natl Acad Sci USA. 2004;101(29):10732‐10737. doi:10.1073/pnas.0404117101 15247411 PMC490003

[8] Louvet L , Lenglet G , Krautzberger AM , et al. Vasorin plays a critical role in vascular smooth muscle cells and arterial functions. J Cell Physiol. 2022;237(10):3845‐3859. doi:10.1002/jcp.30838 35892191 PMC9796581

[9] Luong TTD , Estepa M , Boehme B , et al. Inhibition of vascular smooth muscle cell calcification by vasorin through interference with TGFβ1 signaling. Cell Signal. 2019;64:109414. doi:10.1016/j.cellsig.2019.109414 31505229

[10] Pintus G , Giordo R , Wang Y , et al. Reduced vasorin enhances angiotensin II signaling within the aging arterial wall. Oncotarget. 2018;9(43):27117‐27132. doi:10.18632/oncotarget.25499 29930755 PMC6007470

[11] Sun J , Guo X , Yu P , et al. Vasorin deficiency leads to cardiac hypertrophy by targeting MYL7 in young mice. J Cell Mol Med. 2022;26(1):88‐98. doi:10.1111/jcmm.17034 34854218 PMC8742182

[12] Colombo G , Altomare A , Astori E , et al. Effects of physiological and pathological urea concentrations on human microvascular endothelial cells. Int J Mol Sci. 2022;24(1):691. doi:10.3390/ijms24010691 36614132 PMC9821335

[13] Ni L , Liu L , Zhu W , et al. Inflammatory role of Milk fat globule‐epidermal growth factor VIII in age‐associated arterial remodeling. J Am Heart Assoc. 2022;11(17):e022574. doi:10.1161/jaha.121.022574 36000422 PMC9496444

[14] Wang M , Takagi G , Asai K , et al. Aging increases aortic MMP‐2 activity and angiotensin II in nonhuman primates. Hypertension. 2003;41(6):1308‐1316. 12743015 10.1161/01.HYP.0000073843.56046.45

[15] Wang M , Zhang J , Jiang LQ , et al. Proinflammatory profile within the grossly normal aged human aortic wall. Hypertension. 2007;50(1):219‐227. doi:10.1161/hypertensionaha.107.089409 17452499

[16] Wang M , Zhang J , Spinetti G , et al. Angiotensin II activates matrix metalloproteinase type II and mimics age‐associated carotid arterial remodeling in young rats. Am J Pathol. 2005;167(5):1429‐1442. doi:10.1016/S0002-9440(10)61229-1 16251426 PMC1603787

[17] Wang M , Zhao D , Spinetti G , et al. Matrix metalloproteinase 2 activation of transforming growth factor‐beta1 (TGF‐beta1) and TGF‐beta1‐type II receptor signaling within the aged arterial wall. Arterioscler Thromb Vasc Biol. 2006;26(7):1503‐1509. doi:10.1161/01.ATV.0000225777.58488.f2 16690877

[18] Dean RA , Overall CM . Proteomics discovery of metalloproteinase substrates in the cellular context by iTRAQ labeling reveals a diverse MMP‐2 substrate degradome. Mol Cell Proteomics. 2007;6(4):611‐623. doi:10.1074/mcp.M600341-MCP200 17200105

[19] Prudova A , auf dem Keller U , Butler GS , Overall CM . Multiplex N‐terminome analysis of MMP‐2 and MMP‐9 substrate degradomes by iTRAQ‐TAILS quantitative proteomics. Mol Cell Proteomics. 2010;9(5):894‐911. doi:10.1074/mcp.M000050-MCP201 20305284 PMC2871422

[20] Bonnet AL , Chaussain C , Broutin I , Rochefort GY , Schrewe H , Gaucher C . From vascular smooth muscle cells to Folliculogenesis: what about Vasorin? Front Med (Lausanne). 2018;5:335. doi:10.3389/fmed.2018.00335 30564578 PMC6288187

[21] Korte L , Widmer‐Teske R , Donde K , et al. 13Vasorin controls smooth muscle cell proliferation by regulating EGFR activation. Cardiovasc Res. 2018;114(suppl_1):S3. doi:10.1093/cvr/cvy060.003

[22] Krautzberger AM , Kosiol B , Scholze M , Schrewe H . Expression of vasorin (Vasn) during embryonic development of the mouse. Gene Expr Patterns. 2012;12(5–6):167‐171. doi:10.1016/j.gep.2012.02.003 22426063

[23] Wang M , Jiang L , Monticone RE , Lakatta EG . Proinflammation: the key to arterial aging. Trends Endocrinol Metab. 2014;25(2):72‐79. doi:10.1016/j.tem.2013.10.002 24365513 PMC3917314

[24] Jenkins GM , Crow MT , Bilato C , et al. Increased expression of membrane‐type matrix metalloproteinase and preferential localization of matrix metalloproteinase‐2 to the neointima of balloon‐injured rat carotid arteries. Circulation. 1998;97(1):82‐90. doi:10.1161/01.cir.97.1.82 9443435

[25] McNulty M , Spiers P , McGovern E , Feely J . Aging is associated with increased matrix metalloproteinase‐2 activity in the human aorta. Am J Hypertens. 2005;18(4 Pt 1):504‐509. doi:10.1016/j.amjhyper.2004.11.011 15831360

[26] Wang M , Spinetti G , Monticone RE , et al. A local proinflammatory signalling loop facilitates adverse age‐associated arterial remodeling. PLoS One. 2011;6(2):e16653. doi:10.1371/journal.pone.0016653 21347430 PMC3035650

[27] Chiang HY , Chu PH , Lee TH . MFG‐E8 mediates arterial aging by promoting the proinflammatory phenotype of vascular smooth muscle cells. J Biomed Sci. 2019;26(1):61. doi:10.1186/s12929-019-0559-0 31470852 PMC6716880

[28] Jiang L , Wang M , Zhang J , et al. Increased aortic calpain‐1 activity mediates age‐associated angiotensin II signaling of vascular smooth muscle cells. PLoS One. 2008;3(5):e2231. doi:10.1371/journal.pone.0002231 18493299 PMC2373882

[29] Flavahan S , Chang F , Flavahan NA . Local renin‐angiotensin system mediates endothelial dilator dysfunction in aging arteries. Am J Physiol Heart Circ Physiol. 2016;311(3):H849‐H854. doi:10.1152/ajpheart.00422.2016 27422988 PMC5504433

[30] Miller AJ , Arnold AC . The renin‐angiotensin system and cardiovascular autonomic control in aging. Peptides. 2022;150:170733. doi:10.1016/j.peptides.2021.170733 34973286 PMC8923940

[31] Handa RK , Duckles SP . Influence of age on norepinephrine content in arteries and veins of Fischer 344 rats. Neurobiol Aging. 1987;8(6):511‐516. doi:10.1016/0197-4580(87)90125-4 3431626

[32] Fleg JL , Strait J . Age‐associated changes in cardiovascular structure and function: a fertile milieu for future disease. Heart Fail Rev. 2012;17(4–5):545‐554. doi:10.1007/s10741-011-9270-2 21809160 PMC4677819

[33] Liu G , Hitomi H , Hosomi N , et al. Mechanical stretch potentiates angiotensin II‐induced proliferation in spontaneously hypertensive rat vascular smooth muscle cells. Hypertens Res. 2010;33(12):1250‐1257. doi:10.1038/hr.2010.187 20927110

[34] Wang M , Lakatta EG . Altered regulation of matrix metalloproteinase‐2 in aortic remodeling during aging. Hypertension. 2002;39(4):865‐873. doi:10.1161/01.hyp.0000014506.13322.66 11967241

[35] Zhu N , Guo ZF , Kazama K , et al. Epigenetic regulation of vascular smooth muscle cell phenotypic switch and neointimal formation by PRMT5. Cardiovasc Res. 2023;119(12):2244‐2255. doi:10.1093/cvr/cvad110 37486354 PMC10578915

[36] Wang M , Fu Z , Wu J , et al. MFG‐E8 activates proliferation of vascular smooth muscle cells via integrin signaling. Aging Cell. 2012;11(3):500‐508. doi:10.1111/j.1474-9726.2012.00813.x 22385834 PMC3350574

[37] Schrepfer S , Deuse T , Sultan KR , et al. Inhibition of restenosis development after mechanical injury: a new field of application for malononitrilamides? Cardiology. 2007;108(2):128‐137. doi:10.1159/000096037 17028423

[38] Kawai K , Sakamoto A , Mokry M , et al. Clonal proliferation within smooth muscle cells in unstable human atherosclerotic lesions. Arterioscler Thromb Vasc Biol. 2023;43(12):2333‐2347. doi:10.1161/atvbaha.123.319479 37881937

[39] Wang SS , Li JM , Hu P , et al. Circulating irisin level as a biomarker for pure aortic stenosis and aortic valve calcification. J Cardiovasc Transl Res. 2023;16(2):443‐452. doi:10.1007/s12265-022-10,327-9 36223050 PMC10151307

[40] Rusanov V , Pastushkova L , Luchitskaya E , et al. Potential protein markers associated with the functional state of vessels prior to long‐term space missions and on the first post‐landing day. Acta Astronaut. 2022;195:226‐233. doi:10.1016/j.actaastro.2022.02.020

[41] Nurmohamed NS , Belo Pereira JP , Hoogeveen RM , et al. Targeted proteomics improves cardiovascular risk prediction in secondary prevention. Eur Heart J. 2022;43(16):1569‐1577. doi:10.1093/eurheartj/ehac055 35139537 PMC9020984

[42] Cai Z , Gong Z , Li Z , Li L , Kong W . Vascular extracellular matrix remodeling and hypertension. Antioxid Redox Signal. 2021;34(10):765‐783. doi:10.1089/ars.2020.8110 32460598

[43] Mourino‐Alvarez L , Perales‐Sanchez I , Berna‐Rico E , et al. Association of the Complement System with subclinical atherosclerosis in psoriasis: findings from an observational cohort study. J Invest Dermatol. 2023;144:1075‐1087.e2. doi:10.1016/j.jid.2023.10.031 38036288

